# Near-Infrared Spectroscopy (NIRS) in Traumatic Brain Injury (TBI)

**DOI:** 10.3390/s21051586

**Published:** 2021-02-24

**Authors:** María Roldán, Panayiotis A. Kyriacou

**Affiliations:** Research Centre for Biomedical Engineering, School of Mathematics, Computer Sciences and Engineering, University of London, London EC1V 0HB, UK; maria.roldan@city.ac.uk

**Keywords:** near infrared spectroscopy, traumatic brain injury, cerebral oxygenation, cerebral autoregulation

## Abstract

Traumatic brain injury (TBI) occurs when a sudden trauma causes damage to the brain. TBI can result when the head suddenly and violently impacts an object or when an object pierces the skull and enters brain tissue. Secondary injuries after traumatic brain injury (TBI) can lead to impairments on cerebral oxygenation and autoregulation. Considering that secondary brain injuries often take place within the first hours after the trauma, noninvasive monitoring might be helpful in providing early information on the brain’s condition. Near-infrared spectroscopy (NIRS) is an emerging noninvasive monitoring modality based on chromophore absorption of infrared light with the capability of monitoring perfusion of the brain. This review investigates the main applications of NIRS in TBI monitoring and presents a thorough revision of those applications on oxygenation and autoregulation monitoring. Databases such as PubMed, EMBASE, Web of Science, Scopus, and Cochrane library were utilized in identifying 72 publications spanning between 1977 and 2020 which were directly relevant to this review. The majority of the evidence found used NIRS for diagnosis applications, especially in oxygenation and autoregulation monitoring (59%). It was not surprising that nearly all the patients were male adults with severe trauma who were monitored mostly with continue wave NIRS or spatially resolved spectroscopy NIRS and an invasive monitoring device. In general, a high proportion of the assessed papers have concluded that NIRS could be a potential noninvasive technique for assessing TBI, despite the various methodological and technological limitations of NIRS.

## 1. Introduction

Traumatic brain injury (TBI) is defined as an alteration in brain function or pathology caused by an external force. Based on psychological and anatomical features evaluated by scales such as the Glasgow Coma Scale, TBI can be categorized into mild, moderate, or severe [1]. It is estimated that there are 50 million new cases of TBI every year worldwide [2]. The incidence rate of TBI has increased 3.6% in the last 30 years, and it has been predicted that it will remain the most important cause of disability from neurological disease until 2030, even two to three times higher than the contribution from Alzheimer’s disease [2]. According to the European Union statistics, there are 1.5 million hospital admissions due to TBI per year, with a mortality rate between 30% and 40%, with one person dying every 10 min because of head trauma [2]. The treatment costs associated with TBI are estimated to be around $400 billion annually, resulting in approximately $55,000 per patient [3]. These financial values do not take into account the rehabilitation cost (10% higher than hospitalisation costs) and all the other indirect expenses such as productivity loss, disability, and reduction of quality of life [4].

The most common mechanism of injuries which can cause TBI are hits, falls, violence, concussion, shaken baby syndrome, blast, and whiplash [5,6,7,8,9,10,11]. Patients may suffer unavoidable injuries such as fractures leading to deformation or destruction of brain tissue, whereas avoidable secondary injuries such as swelling and hematomas, which compress vital brain structures and displace the brain midline, may also take place. In many cases, those secondary injuries are followed by obstructions of cerebrospinal fluid (CSF) paths—generating hydrocephalus—as well as blockage of arteries which may result in ischemia. These damages can also alter autoregulation capacity, increase intracranial pressure, and reduce blood pressure to the brain, which, in turn, can all lead to brain hypoxia [12,13].

Considering that secondary brain injuries often take place within the first hours after a trauma, noninvasive monitoring might be helpful in providing early information on the brain’s condition [14]. Unfortunately, continuous and noninvasive monitoring of brain’s hemodynamics is scarcely available, even for severe cases of TBI [15]. Current clinical practices primarily utilize invasive methods for assessing TBI. Such methods include probes and/or catheters to measure intracranial pressure (ICP), brain temperature (BT), brain oxygen tension (PbtO_2_), neurochemistry via microdialysis (MD), cerebral blood flow (CBF), and jugular oxygen saturation (SjvO_2_) [16]. The aforementioned approaches introduce additional risks and require neurosurgical expertise, thus potentially causing delays in providing useful clinical information [17,18]. Furthermore, studies have shown that an initial phase of cerebral hypoperfusion, which is not assessed and treated on time during the early post-traumatic period, can contribute to increased mortality and worsened neurological outcome in 60–80% of TBI patients [15,18]. Imaging techniques such as computerized tomography (CT) and magnetic resonance imaging (MRI) could provide a solution to the above challenges, but such techniques cannot be used for continuous monitoring of TBI at the bed site [19].

In consequence, an ideal neuromonitoring system should be continuous, affordable, noninvasive, and suitable for bedside use or in field monitoring (i.e., ambulances) [20]. Since 1977, when near-infrared spectroscopy (NIRS) was first described for monitoring cerebral perfusion and brain oxygenation [21], clinical interest on this optical technology has increased. Near-infrared (NIR) represents wavelengths within the range of 700 nm and 1000 nm, where the absorption contribution of chromophores such as oxygenated and deoxygenated hemoglobin is maximized, while the absorption contribution of other compounds such as water molecules is minimized. NIR light can penetrate bony structures and several millimetres into cerebral tissue, where according to Beer–Lambert law, light absorption is directly proportional to the concentration of chromophores. The reflected light attenuation represents information regarding regional cerebral oxygen saturation (rSO_2_) and the balance between oxygen delivery and oxygen consumption, making NIRS a very sensitive technology to changes in cerebral oxygenation [22]. Based on the aforementioned, NIRS could potentially address ideal neuromonitoring requirements, detect brain tissue at risk of secondary injury, and complement or even replace current invasive practices [23,24]. Overall, current evidence suggests that NIRS allows the detection of intracranial bleeding, the assessment of brain tissue oxygenation and cerebral perfusion. NIRS has also been applied in the evaluation of cerebral autoregulation and intracellular metabolic state during the early post-traumatic period. Furthermore, this optical technique can be also applied during neurorehabilitation [23].

This review aims to rigorously investigate the utilisation of NIRS in TBI and provide a synthesis of the available evidence of the association between continuous NIRS-based measurements and commonly monitored neurophysiological parameters, such as oxygenation and cerebral autoregulation. So far, no review is known by the authors that synthesized multiple clinical applications of NIRS in TBI nor considered the different types of NIRS technologies in relation to TBI physiology. This synthesis highlights several factors that are important for future research, including literature trends, the comparison between NIRS measurements and validated neuromonitoring parameters, and their relationship with brain physiological changes following trauma.

## 2. Materials and Methods

**Figure 1:**
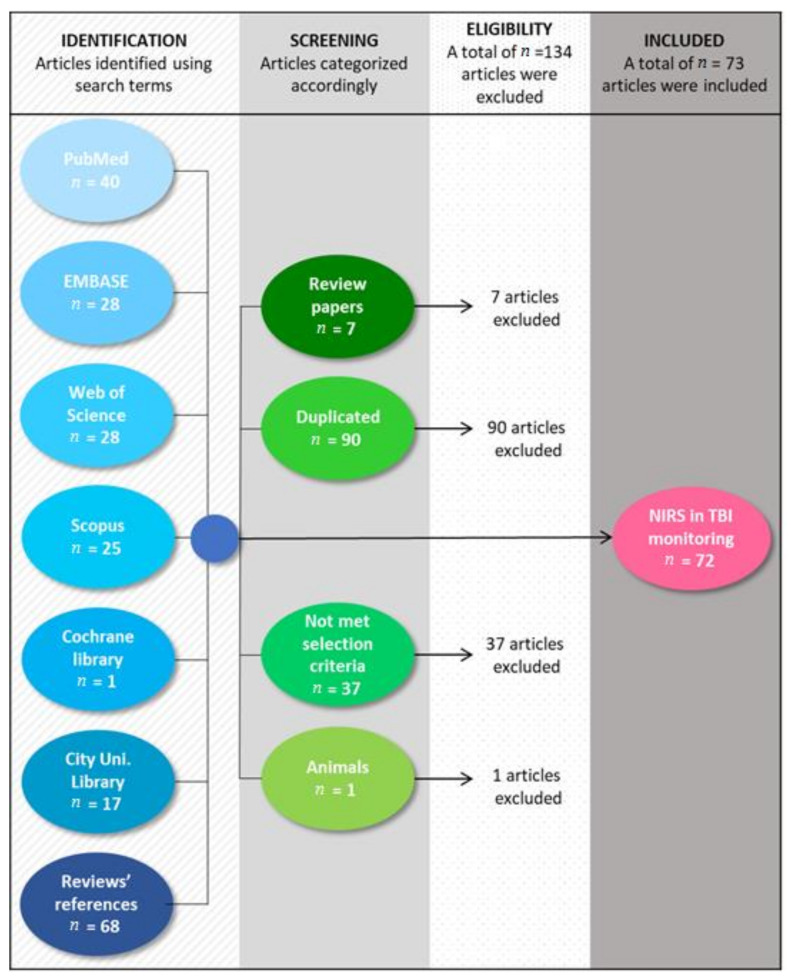
Flowchart of the methodology used to include 72 out of 207 studies published until July 2020.

PubMed, EMBASE, Web of Science, Scopus, and Cochrane library databases were used to search for literature that investigates the use of NIRS in TBI monitoring. As this review aims to describe every technological advancement of NIRS in TBI monitoring, the studies were included without time restriction. The search terms used were a combination of the terms “near-infrared spectroscopy,” “NIRS,” “TBI,” and “traumatic brain injury”, assessed on the title, abstract, and keywords. Databases search yielded 139 results, seven of which were review papers. The references from these reviews were examined, resulting 68 publications of interest for the current research. In total, the search resulted 207 papers; 90 of which were duplicates and were removed. From the remaining 117 papers, 72 met the inclusion criteria and were included in the analysis. Papers were included in the review if they used NIRS in TBI monitoring and were written in English. Review articles, nonoriginal, nonindependent documents and animal studies were excluded (see Figure 1). It is recognized that a potential limitation of this review is the possibility of search engine bias. Only articles that were tagged with these terms were included and therefore, qualifying articles may have been missed in the search.

Demographics, study characteristics, and outcome measures were extracted from the selected articles and compiled in an electronic database. Data fields included bibliographic data, sample size, patients’ characteristics, NIRS application, outcome measures, limitations, and conclusions. Papers were classified into five categories according to NIRS applications: diagnosis, prognosis, treatment, correlation, and comparison between NIRS technologies capacities. The diagnosis group was also subdivided into subgroups relating to the outcome measured: oxygenation, autoregulation, hematomas, and neurorehabilitation. Oxygenation, autoregulation, and hematomas monitoring are relevant during TBI’s “golden hour”, while neurorehabilitation monitoring usually takes place some months after injury. Moreover, oxygenation and autoregulation monitoring utilize similar NIRS sensors and algorithms, while hematomas monitoring involves multiple NIRS sensors and a specific algorithm for hematoma detection. Thus, this review examined the main applications of NIRS for TBI monitoring, and it presents a thorough revision of those applications on oxygenation and autoregulation for TBI patients’ diagnosis.

## 3. Results

### 3.1. Search Results

**Figure 2:**
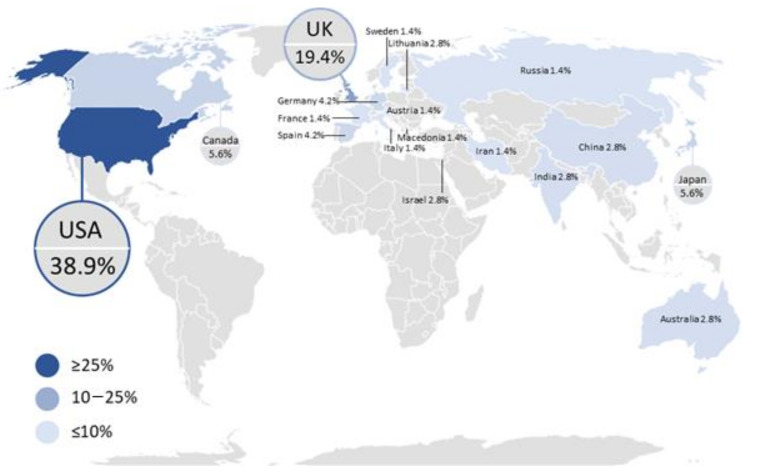
Global distribution of scientific articles that discussed the use of near-infrared spectroscopy (NIRS) technology in traumatic brain injury (TBI) monitoring until July 2020. The number of publications per country is indicated by the intensity of the color, with darker colors representing a higher number of articles than lighter colors.

The literature search returned 72 scientific publications that met the inclusion criteria, all of which were included in the general analysis of NIRS application in TBI monitoring. To gain a better understanding of the trends and geographical distribution of the included papers, the data were analyzed by year and country as well as by NIRS applications and outcome measure. The distribution of the published literature by country indicates that the United States of America (USA) provided the most significant contribution in the field with 28 publications, followed by the United Kingdom (UK) with 14 publications, as shown in Figure 2. With the USA as the front runner in terms of volume of research, it is recommended that other countries continue or increase their investigations in this field, given the substantial impact of TBI on mortality and disability rates [7]. This recommendation is also justified by the fact that 80% of the incident cases per year are from low and middle income countries [2], while most of the scientific contribution is from high income countries. As this research intended to describe the characteristics and advantages of NIRS in TBI monitoring for its future use across various populations, ideally NIRS should be assessed across individuals of various ethnic backgrounds from around the world.

**Figure 3:**
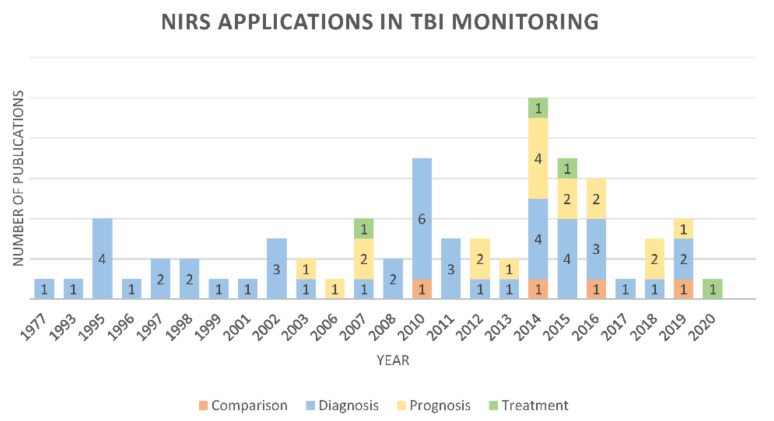
Number of publications per year associating TBI with the various types of NIRS applications.

**Figure 4:**
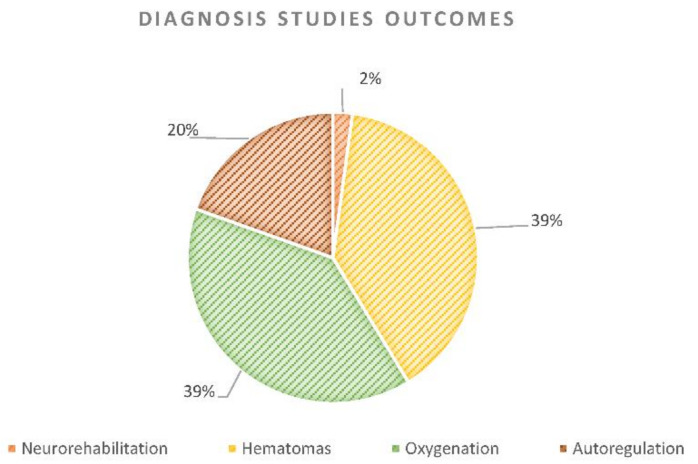
Percentage of diagnosis papers per subgroup according to the outcome measured: oxygenation, autoregulation, hematomas, and neurorehabilitation monitoring.

Until July 2020, the number of papers published that have used NIRS in TBI monitoring experienced a downward trend. The largest amount of publications on the topic were in 2014. However, since 2003, other applications in addition to diagnosis have been reported (Figure 3). In total, 46 (64%) publications were found that used NIRS as a diagnostic tool in TBI monitoring. Figure 4 shows the percentage of diagnosis papers per subgroup. The outcome of neurorehabilitation is mainly related to prefrontal cortex features. All the prognosis papers (*n* = 18) assessed patients’ neurorehabilitation using NIRS, however, there was just one diagnosis publication where NIRS was used to identify regions within the prefrontal cortex that contributed to distinguishing between TBI and healthy subjects [25]. Likewise, 18 studies were included that explored NIRS accuracy for hematoma detection after trauma. Hematoma detection using NIRS technology is based on differential light absorption of the left versus the right side of the brain [26,27,28,29]. Brain absorption is symmetrical under normal circumstances and when extravascular blood is present due to a hematoma, the reflected component of light is significantly lower [26,27,28,29]. The absorption differential can be detected via NIRS units placed over symmetrical locations on both sites of the head [26,27,28,29]. Neurorehabilitation and hematomas monitoring are relevant outcomes in TBI monitoring, however, the current research presents a thorough revision of NIRS applications on oxygenation and autoregulation due to the relevance of those outcomes in TBI’s “golden hour” and their technological similarities [30,31].

### 3.2. Physical and Technological Description of NIRS

Red blood cells (RBC) contain a protein with high gas-bounding capacity called hemoglobin, which allows oxygen transport [32,33]. When gas exchange takes place in the lungs, each hemoglobin binds to four oxygen molecules. Then, RBCs carry this oxyhemoglobin (HbO_2_) through the arteries to the tissues, where oxygen is delivered through a process known as oxygen perfusion. Although some oxygen can still be bound to hemoglobin, when it has released most of the oxygen molecules (deoxyhemoglobin HHb), it can collect part of the CO_2_ discharged by the tissues. Finally, CO_2_ is transported in venous blood and is released into the lungs, where it is expelled by exhalation [32,33].

**Figure 5:**
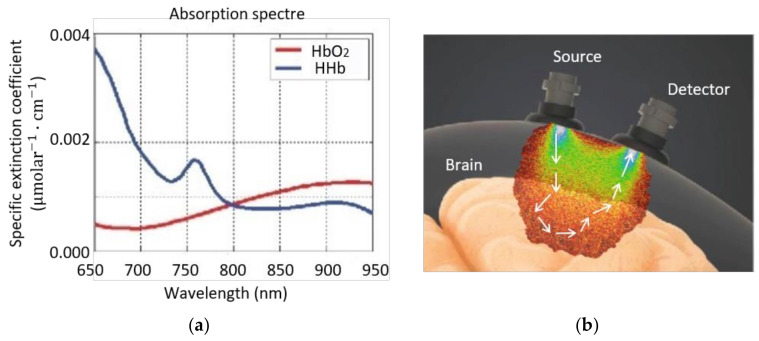
(**a**) Absorption coefficient spectra of oxyhemoglobin (HbO_2_) and deoxyhemoglobin (HHb). (**b**) The probabilistic trajectory of photons from the source to a detector of incident near-infrared light is described as a “banana shape” [38]. Figure modified from [39].

Arterial blood, rich in O_2_, has a bright red color, while venous blood color is dark red due to its poor O_2_ content. This physical property is used by near-infrared spectroscopy (NIRS) as explained below [32]. Near-infrared (NIR) light is absorbed to different degrees by the chromophores, oxyhemoglobin, deoxyhemoglobin, and cytochrome-c-oxidase, at wavelengths near 700–1300 nm [34]. Within this range, light has low absorption and high scattering properties, causing the light to go deeper into the tissue, therefore enabling real-time noninvasive monitoring of brain tissue oxygen saturation [21]. NIRS uses the different absorption properties of these chromophores to quantify their concentrations in tissues [35]. NIRS bases its principles on the Beer–Lambert law, which correlates the absorption of light passing through the tissue to the absorption coefficient ($\mu_{a}=\varepsilon \cdot c$) and the pathlength (l) travelled by light (Equation (1)) [36].

(1) $$A=\ln\frac{I_{0}}{I}=\varepsilon \times c\times l$$

where *A* is light attenuation, also known as optical density, *I* is transmitted light intensity, *I*_0_ is incidence light intensity, ε is the tissue’s extinction coefficient (Figure 5a), *c* is chromophore concentration and *l* is distance travelled by light in the tissue on the assumption that light only undergoes absorption [37].

When the tissue contains multiple chromophores, total light attenuation (A) can be defined as the linear sum of the contribution of each chromophore’s concentration. Thus, the tissue is illuminated with as many wavelengths as chromophores are being assessed. The result is a system of equations with the specific purpose of calculating the concentrations of HbO_2_ and HHb by NIRS [40]:

(2) $$A_{\lambda_{1}}=\left(\varepsilon_{{\mathrm{HbO}}_{2}}{}_{\lambda_{1}}\times \left[{\mathrm{HbO}}_{2}\right]+\varepsilon_{\mathrm{HHb}}{}_{\lambda_{1}}\times \left[\mathrm{HHb}\right]\right)\times l$$

(3) $$A_{\lambda_{2}}=\left(\varepsilon_{{\mathrm{HbO}}_{2}}{}_{\lambda_{2}}\times \left[{\mathrm{HbO}}_{2}\right]+\varepsilon_{\mathrm{HHb}}{}_{\lambda_{2}}\times \left[\mathrm{HHb}\right]\right)\times l$$

where $\lambda_{1}$ and $\lambda_{2}$ are two different wavelengths. However, this assumption is not completely valid in tissues, where near-infrared light attenuation (A) is highly dominated by scattering (roughly 80% scattering vs. 20% absorption) [37]. Scattering in tissue causes an increase of the pathlength travelled by light, as well as the loss of scattered light because it cannot reach the detector (Figure 5b). The modified Beer–Lambert law considers the effects of scattering in tissues [37,41], where the distance travelled by light (l) is replaced by the product of the differential path factor (DPF) and the separation distance (d) between the sensor’s emitter and detector [41], as is shown in Equation (4). The DPF is the increase on the light’s pathlength due to scattering, where this coefficient depends on the wavelength, tissue type, and emitter detector distance [38]. Finally, the modified Beer–Lambert law adds the factor *G*, or scattering coefficient ($\mu_{s}$), to the equation, which represents both the nature and the geometry of the tissue [41].

(4) $$A_{\lambda }=\left(\varepsilon_{1}{}_{\lambda }\times \left[C_{1}\right]+\varepsilon_{2}{}_{\lambda }\times \left[C_{2}\right]+\ldots \varepsilon_{n}{}_{\lambda }\times \left[C_{n}\right]\right)\times d\times \mathrm{DPF}+G$$

Although increasing emitter–detector separation distance increases penetration depth, it also causes a decrease in overall signal quality due to high absorption [42]. Thus, emitter–detector separation distances between 2.5 and 5.0 cm is the range suggested to obtain good quality NIRS measurements [35,38].

Two of the major parameters of interest in perfusion analysis are tissue oxygen saturation index (rSO_2_) and tissue oxygenation index (TOI). Both represent the percentage of oxygenated hemoglobin in the sample volume [43]. Near-infrared spectroscopy is used for the determination of changes in HbO_2_ and HHb concentrations. From these data, some commercial NIRS systems derive rSO_2_ (i.e., INVOS 5100 (Medtronic, MN, USA)). Other sensors interrogate the change of light attenuation along the detector’s distance to calculate the relative concentrations of HbO_2_ and HHb and generate TOI (i.e., NIRO-300 (Hamamatsu Photonics KK, Hamamatsu City, Japan)). Despite the differences of the algorithms, both indices are expressed as percentages of oxygenated hemoglobin relative to total hemoglobin [44], as is shown in Equation 5.

(5) $$\frac{\left[{\mathrm{HbO}}_{2}\right]}{\left[{\mathrm{HbO}}_{2}\right]+\left[\mathrm{HHb}\right]}\times 100\%$$

NIRS cannot discriminate between arterial blood, capillaries, and venous blood. In consequence, oxygen saturation measured by NIRS represents a mixed saturation between arterial and venous blood. This measure is predominantly from venous oxygenation, as approximately 75% of cerebral blood is venous [45]. The above explains why NIRS measurements are always lower than arterial oxygen saturation measured by pulse oximetry (SpO_2_), which assesses only arterial blood by utilizing the arterial pulsatile component of the signal [37].

### 3.3. NIRS Measurement Techniques

**Figure 6:**
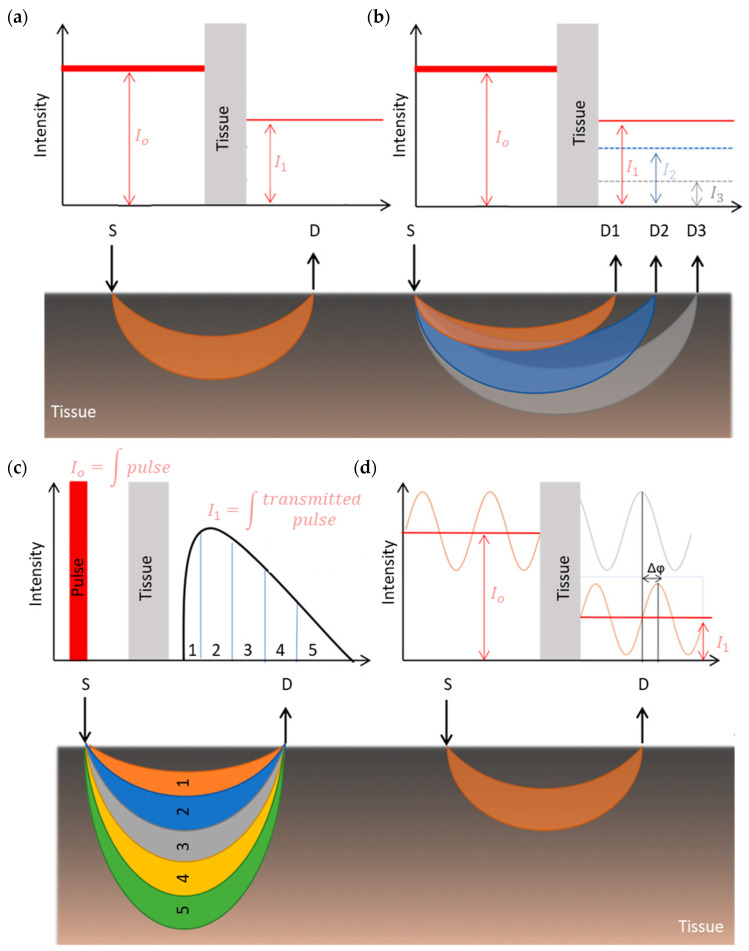
Schematic diagram of NIRS detection modes. (**a**) Continuous wave NIRS (CW-NIRS), (**b**) spatially resolved spectroscopy (SRS), (**c**) time-resolved spectroscopy (TD-NIRS), and (**d**) phase modulated spectroscopy (PMS). The figure also shows a representation of the photon path in tissues for each technique. S: source, D: detector. (Figure modified from [49]).

Four measurement techniques have been developed since NIRS was invented. All of them aim to quantify HbO_2_ and HHb concentration by applying different measurement principles [36], as shown in Figure 6.

In continuous wave NIRS (CW-NIRS), as the name suggests, light of constant intensity is “injected” into tissue, and then the attenuated light signal is measured at a distance from the light source (Figure 6a). This technique assumes that HbO_2_ and HHb are the only absorbers in tissue. It also assumes that scattering is constant during the entire measurement (ΔG = 0) and DPF is considered to be between one half and one third of the actual emitter–detector separation distance. In consequence, the modified Beer–Lambert law (MBL) is used to calculate the change in chromophores concentrations [35,38,46,47]. However, its major challenge is to remove any superficial layer contamination from the brain signal, taking into account that the brain is covered by multiple layers which are perfused by vessels that carry hemoglobin, leading to extracerebral signals that play a contaminating role on CW-NIRS [48].

Spatially resolved spectroscopy (SRS) is based on the measurement of light attenuation at several source–detector separations (Figure 6b), where relative concentrations of HbO_2_ and HHb can be calculated by solving the diffusion equation as a function of the photodetectors’ distance [36,41,50,51]. This method enhances the contribution of deeper tissues (S-D2 or S-D3) while reducing the contribution of more superficial tissues (S-D1). The latter is the biggest advantage of the SRS design over the MBL algorithm, when using CW-NIRS. Time-resolved spectroscopy mode (TD-NIRS), uses short light pulses to illuminate the tissue, where the scattered photons take more time to be detected than the unscattered photons (Figure 6c). Average photons’ arrival time allows the calculation of the light pathlength, therefore enabling the calculation of the chromophores’ absolute concentrations by solving the diffusion equation [36,41,46,47]. In phase modulated spectroscopy (PMS), the emitted light is modulated in frequency and intensity, thus, measured attenuations and frequency shift are used to estimate the light pathlength and the absorption and scattering properties of the tissue (Figure 6d). Once these are derived, at least from two wavelengths, absolute concentrations of HbO_2_ and HHb can be calculated from the modified Beer–Lambert law [36,41,46,47].

### 3.4. Subgroups

The previous sections described the principles of NIRS. Many clinical and research applications using NIRS in TBI monitoring have been reported in the last 40 years. This section provides an overview of its application in oxygenation and autoregulation monitoring for TBI diagnosis, considering different types of NIRS technologies and TBI’s physiology.

#### 3.4.1. Oxygenation

##### Physiology of Oxygenation in TBI

The brain is dependent on continuous oxygenated blood supply. Ten seconds of ischemia leads to unconsciousness, 20 s ceases neuronal activity, and a few minutes of ischemia lead to irreversible damage [52]. The internal carotid system provides 80% of cerebral blood, where each side supplies an ipsilateral cerebral hemisphere [52]. Once blood reaches the capillary bed, oxygen and nutrient delivery are exchanged for disposable substances such as carbon dioxide (CO_2_) This deoxygenated blood is drained by two sets of veins, the superficial and deep veins, which join the cerebral sinuses. Then, deoxygenated blood reaches the internal jugular vein, which returns blood to the right atrium of the heart [52,53].

**Figure 7:**
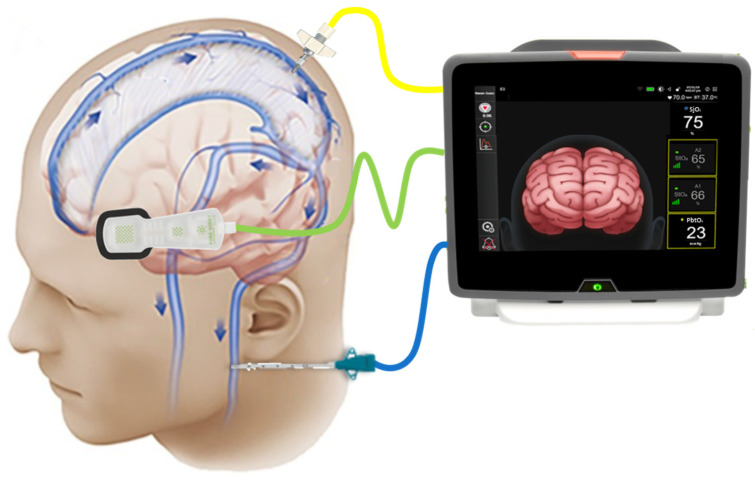
NIRS-derived regional tissue oxygen saturation (green probe) analyzes the oxygen concentration in cerebral microcirculation, which is predominantly from venous oxygenation. Jugular bulb venous saturation (blue probe) analyzes the remaining oxygen concentration that flows from the venous sinuses to the internal jugular vein. Both are indirect measurements of how much oxygen is being used by the brain. Brain tissue oxygenation tension (yellow probe) analyzes the dissolved oxygen within the cerebral plasma that diffuses across the blood brain barrier (BBB). (Figure modified from [57,58]).

NIRS-derived regional cerebral oxygen saturation (rSO_2_) is an indirect marker of cerebral venous oxygenation and has been shown to correlate with jugular bulb venous saturation (SjO_2_), see Figure 7 [54]. As it was explained above, deoxygenated blood and the remaining oxygen flows through the internal jugular veins to return to the heart. Therefore, the measurement of SjO_2_ helps establish the balance between cerebral blood flow (CBF) and metabolic requirement (CMRO2), giving an indication of the use of oxygen by the brain [55]. Accordingly, when oxygen demand increases, brain extracts a greater amount of oxygen, resulting in decreased jugular venous oxygen saturation. Conversely, when CBF exceeds metabolic requirement, venous oxygen saturation is higher [55]. The following equation explains the physiology behind SjO_2_ and its dependency on CBF and CMRO2, assuming constant arterial oxygenation (SaO_2_) [55,56].

(6) $${\mathrm{SjO}}_{2}={\mathrm{SaO}}_{2}\;-\;({\mathrm{CMRO}}_{2}/\left(1.34\left[\mathrm{Hb}\right]\times \mathrm{CBF}\right))$$

After TBI, the coupling between CMRO2 and CBF is lost. Thus, intracranial hypertension leads to brain hypoperfusion, which is not accompanied by a proportional reduction in CMRO2. Consequently, SjO_2_ falls from a normal 60–75% range to values below 55%, which are associated with a poor outcome. SjO_2_ might decrease below 50% when at least 13% of the brain has become ischemic, due to oxygen supply being critically low for metabolic demand [12,55]. Likewise, when cerebral autoregulation fails because of trauma, the vasodilatory response increases CBF, leading to cerebral hyperemia with decreased oxygen extraction. Therefore, SjO_2_ values rise over 75%, representing an unbalance between CBF and CMRO2 [55]. Likewise, a NIRS-derived rSO_2_ value less than 60% for extended periods after TBI is associated with higher mortality, intracranial hypertension, and impaired cerebral perfusion. Also, rSO_2_ is moderately accurate at predicting severe brain hypoxia [54].

Another fundamental pathophysiological consequence after TBI is an imbalance of oxygen delivery to neural tissue [59]. Understanding the physiology of brain tissue oxygen tension (PbtO_2_) is significantly important in this research. PbtO_2_ reflects the dissolved oxygen within the plasma that diffuses across the blood-brain barrier rather than the entire oxygen content or cerebral metabolism [60]. The above, due to PbtO_2_, is significantly related to the product of CBF and arteriovenous oxygen tension difference (Equation (7)), which is influenced by the oxygen diffusion gradient [56,60,61]. Equation (7) presents the association between PbtO_2_ and oxygen diffusion or cerebral blood flow, where the conversion factor 1 mmHg = 0.0031 mL/100 gr can be used to keep Equation (7) dimensionally consistent in millimeter of mercury [56]. Thus, PbtO_2_ is not simply an ischemia biomarker, since several variables might modify oxygen diffusion or metabolism, even under normal CBF conditions [61]. However, low PbtO_2_ values can result from low PaO_2_, local O_2_ extraction impairment, or decreased cerebral blood flow [60,61].

(7) $${\mathrm{PbtO}}_{2}\;\approx \mathrm{CBF}\times \left({\mathrm{PaO}}_{2}-{\mathrm{PjO}}_{2}\right)$$

PbtO_2_ values around 23–35 mmHg are typically found in a healthy state. Values below 20 mmHg are considered abnormal and have been associated with greater evidence for cerebral ischemia and energy dysfunction. Some authors suggest treatment when PbtO_2_ is below 15 mmHg [61]. Thresholds for ischemia are not yet clearly defined, but a PbtO_2_ below 8–10 mmHg seems to indicate a high risk of ischemia in patients with subarachnoid hemorrhage. Low values of PbtO_2_, particularly if they are sustained, are associated with poor outcome after traumatic brain injury, and there is some evidence that brain tissue oxygen-directed therapy may improve outcome in such patients [45].

As it has been explained above, oxygen supply is a key component in secondary cerebral damage, nevertheless, it is also important to consider cellular metabolism failure as a possible outcome after trauma. The latter inhibits the use of delivered oxygen and glucose by the mitochondria, hampering adenosine triphosphate (ATP) synthesis. Cytochrome-c-oxidase concentration (oxCCO) is a chromophore involved in mitochondrial oxygen delivery and utilization, which makes it also a possible noninvasive biomarker for TBI monitoring.

The following subsections provide a concise description of the experimental results as presented in the reviewed papers, their interpretation, as well as their drawn conclusions.

##### Summary of the Evidence on NIRS-Derived Oxygenation in TBI

The literature search returned 18 scientific publications that assessed NIRS-derived oxygenation in TBI patients. Table 1 summarizes the main characteristics of the methodology and results presented in each paper. The documents were sorted chronologically as is shown in the column of author and year of publication. Moreover, sample size (*n*) is reported with the proportion of male and female (m:f) included in the studies. Table 1 also includes the type of NIRS devices used, the manufacturer, and the main technical specifications. Additionally, all the comparisons between NIRS output and the control measurements were extracted from the papers. In four cases, the comparison was between the outcome of the basal condition and after an intervention (i.e., hyperventilation, MAP changes). Finally, the main conclusion of each author is shown in the last column of the table.

##### Sample Size and Patient Demographics

**Figure 8:**
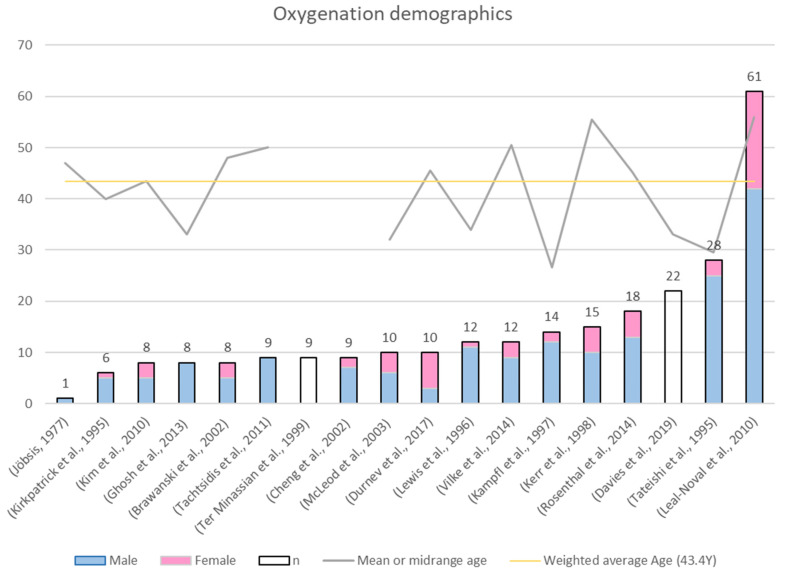
Demographics of the evidence on NIRS-derived oxygenation in TBI.

This review identified 17 publications spanning between 1995 and 2019 where NIRS was used for oxygenation monitoring in TBI patients. The only publication that was included in this review without meeting the inclusion criteria was Jöbsis publication. This exception was made due to Jöbsis’s pioneering work being the first approach to NIRS application in vivo, which demonstrated that oxygen sufficiency can be monitored noninvasively using NIR light [21]. Moreover, Figure 8 shows that 75% of the studies had a sample size smaller than 15 patients, which is a limitation in the results of almost all these publications. Likewise, it was not surprising that in 15 of the 16 papers that included the sex ratio, the number of men was significantly higher than the number of women. Literature has always reported a proportion of males greater than that of females [1], where men have more than double the risk of TBI than women [2]. Finally, the mean or midrange age (grey line) was stated in 16/18 papers, therefore, a weighted average of 43.4 years was calculated (yellow line). Interestingly, all studies included mostly middle-aged people, who are at risk of suffering severe TBI but can also be rapidly stabilized in intensive care.

##### Distribution of NIRS Techniques

**Figure 9:**
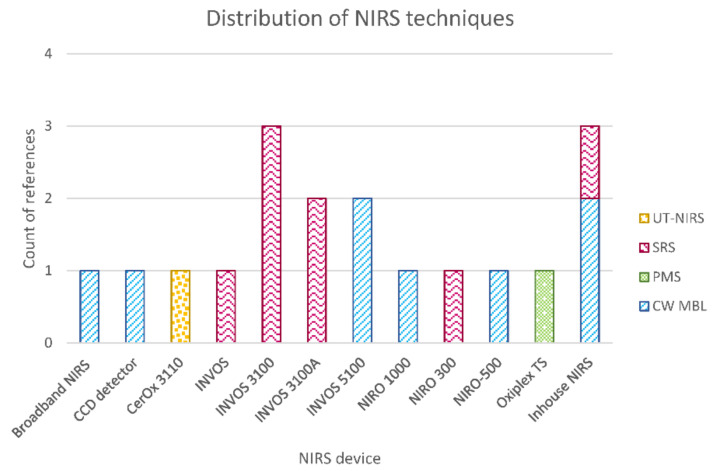
Distribution of NIRS techniques used for oxygenation monitoring in TBI patients. Where continuous wave modified Beer–Lamber law (CW MBL), spatially resolved spectroscopy (SRS), phase modulated spectroscopy (PMS), and ultrasound-tagged near-infrared spectroscopy (UT-NIRS).

Figure 9 shows the distribution of NIRS techniques used for oxygenation monitoring in TBI patients. As it was expected, the most-used technique was continuous wave (CW) NIRS, analyzed by either the modified Beer–Lambert law or spatially resolved spectroscopy (SRS) algorithms. The latter considered source–detector (S-D) distances between 2.5 and 6 cm in reflectance mode. Jöbsis is the only author who utilized transmission mode with an S-D distance of 13.3 cm. Time-resolved spectroscopy has not been reported in TBI monitoring yet, as nowadays this technique is mainly used in research laboratories [39]. Davies et al. reported the only paper included in this review, which compared phase modulated spectroscopy with the invasive measurement of brain tissue oxygenation tension [78]. Finally, Rosenthal et al. used a novel technique called ultrasound-tagged near-infrared spectroscopy (UT-NIRS), which is a hybrid technology that induces an artificial modulation in the detected light intensity by applying ultrasound waves. In this manner, only light from a specific volume of brain tissue is selected for analysis [75].

##### Appropriate Clinical Comparable Parameters

**Figure 10:**
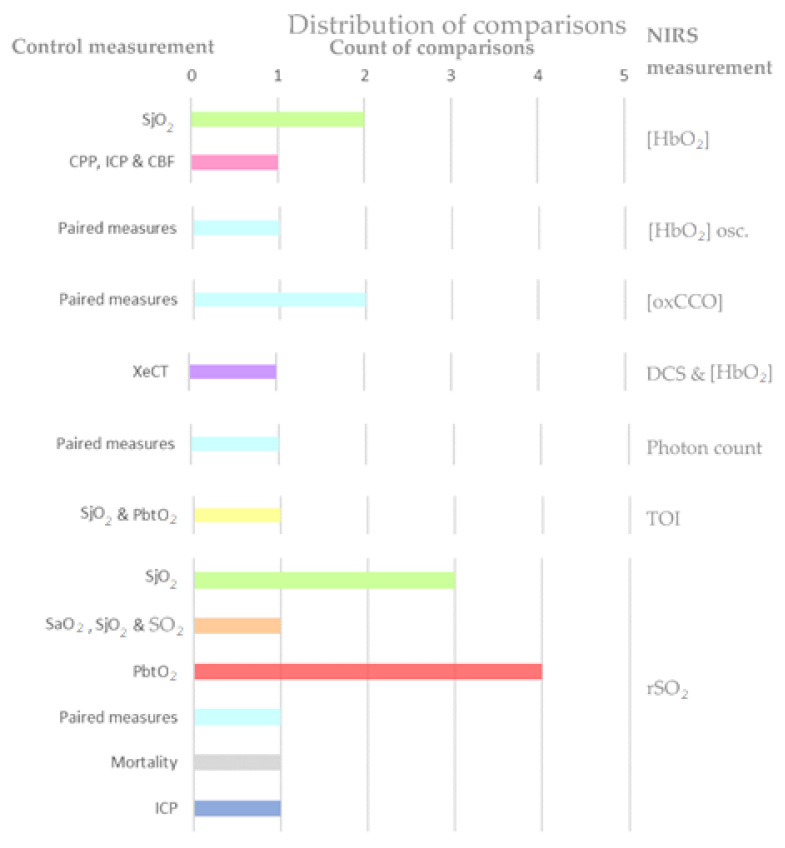
Comparisons found in the evidence on NIRS-derived oxygenation in TBI.

Different comparisons have been done in the last few years to assess the capability of NIRS in oxygenation monitoring. Primarily, five papers have compared NIRS-derived measurements before and after an intervention. Jöbsis compared the amount of photons detected by the photodetector at different stages of hyperventilation [21], while Tachtsidis et al. and Ghosh et al. assessed the variations in oxidized–reduced cytochrome c oxidase (oxCCO) concentration before and after changing PaCO_2_ and FiO_2_, respectively [73,74]. Likewise, Durnev et al. correlated rSO_2_ with changes in MAP, while Cheng et al. assessed the signal oscillations present in hemoglobin concentration [68,77]. The distribution of these paired measurements is shown in light blue in Figure 10.

On the other hand, independent comparisons were found between the NIRS-dependent outcome and a group of physiological variables. In these evaluations, each variable of the control group was not compared with NIRS outcome directly. In Kirkpatrick’s paper, ICP, CPP, and relative CBF changes were not compared individually with hemoglobin concentration, yet it was reported that NIRS only registered obvious changes when the control group varied [63]. Similarly, McLeod et al. did not assess the correlation between TOI and invasive measurements, however, altering the fraction of inspired oxygen, each cerebral oxygenation variable changed significantly [70]. Furthermore, Kerr et al. used a regression model to define rSO2 as a function of the control variables SaO_2_, SjvO_2_, and extracranial SO2. The coefficients in this model were the weights of each control variable adjusted by all the parameters included in the regression [66].

Finally, independent comparisons were reported between NIRS measurements and reference variables such as SjvO_2_, PbtO_2_, ICP, or XeCT. The latter was only used by Kim et al. to examine the correlations between changes in NIRS parameters and changes in CBF, determined by XeCT [71]. Another specific comparison that was reported only in one paper was done by Kampfl et al., who evaluated rSO_2_ values in patients with high and low ICP (threshold = 25 mmHg) after hyperoxygenation [65]. Conversely, invasive oxygenation parameters SjvO_2_ and PbtO_2_ were widely used as reference variables or gold standard measurements. Both variables’ distributions are shown in Figure 10 in green and red, respectively. Jugular venous oxygen saturation monitoring using a fiberoptic catheter is the method that is currently accepted as a technique for continuous measurement of cerebral oxygenation [56,62,63,64,67]. However, it entails technical difficulties, including a relatively small amount of time for collecting good quality data and the need for frequent recalibrations [75]. Also, as NIRS measurements represent the average value of arterial, capillary, and venous blood, there can be a difference in the time course of oxy- and deoxyhemoglobin from that in SjO_2_ [62]. On the other hand, PbtO_2_ is not yet an accepted gold standard technique for cerebral monitoring, although some studies used it as such [56,69,72,79]. Simultaneous comparison of PbtO_2_ and rSO_2_ is problematic, as each monitor utilizes a distinct physical principle and measures a distinct physiological parameter [72].

#### 3.4.2. Autoregulation

##### Physiology of Autoregulation in TBI

Autoregulation is the capacity of cerebral circulation to maintain a continuous and independent CBF and adequate oxygen supply despite changes in blood pressure, cerebral perfusion pressure, hematocrit, blood viscosity, and partial pressure of arterial oxygen and carbon dioxide [80,81]. Multiple physiological processes are involved in cerebral autoregulation, where the myogenic mechanism relies on the cerebrovascular reactivity (CVR), which can provoke either vasodilatation or constriction. A decrease of mean arterial pressure (MAP) or cerebral perfusion pressure (CPP) causes cerebral vasodilatation, while an increase of MAP or CPP leads to vasoconstriction. [82]. However, CVR is not exclusively associated to variations in pressure; changes in other physiological processes such as CO_2_ or O_2_ reactivity, mediated by activation of nitric oxide, H^+^ ions, and other metabolites in the arterial endothelium, can also lead to a rapid cerebral vasomotor response which regulates CBF [82,83]. The latter is known as the metabolic mechanism, and it may be initiated by hypoxia, dehydration, or hypercapnia [80]. Finally, the neurogenic mechanism focuses on neurotransmitter-mediated changes in vascular tone, originated by fluctuations in the sympathetic and parasympathetic system [81]. For instance, the trigeminal-cerebrovascular system plays a central role in counteracting vasoconstriction through the production of calcitonin gene-related peptide (CGRP), a potent vasodilator [81,84].

**Figure 11:**
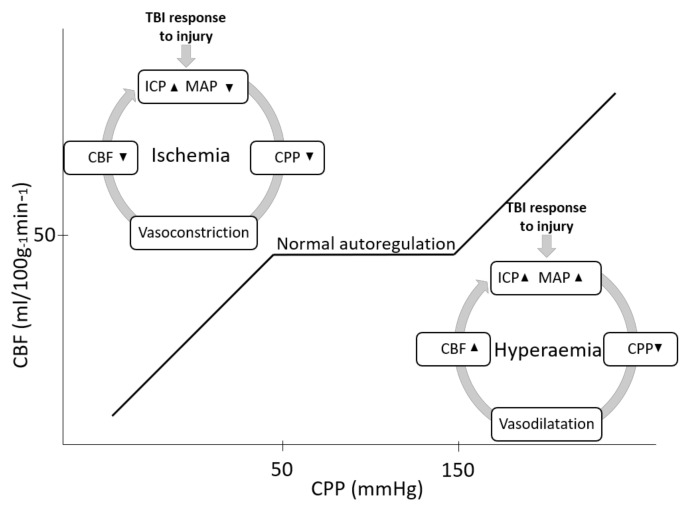
Cerebral autoregulation, where cerebral blood flow is maintained constant over a range of MAP/CPP. Out of the autoregulatory plateau, CBF becomes pressure-dependent and intracranial pressure dangerously rises (modified from [80,83,85,86]). Hypotension with disrupted cerebral autoregulation rapidly leads to cerebral ischemia, while hypertension above the autoregulatory threshold increases the risk of hyperemia.

When autoregulation is intact, suitable coupling is observed between a small rise in CBF and metabolism [80]. In most cases, autoregulation pressure lies in the range of 50–150 mmHg [85]. In chronic arterial hypertension, autoregulation limits are displaced to higher levels, shifting the curve to the right as high as 40 mmHg [85]. Another characteristic of this protector mechanism is its temporal response, which occurs within two to ten seconds after a sudden change in MAP/CPP [83]. Figure 11 represents the behavior of relevant hemodynamic variables in the function of CBF. It depicts that out of the autoregulatory plateau, CBF becomes pressure-dependent, resulting in too much or too little blood perfusion to the brain [80].

After brain trauma, cerebral hemodynamic variables change as a response to secondary injuries like hemorrhages, edema, or BBB disruption. In accordance, blood pressure might drop by substantial blood loss due to extracranial injuries. Significant hemorrhage decreases stroke volume and cardiac output, causing the body to compensate through systemic vasoconstriction [87,88]. Thus, hypotension leads to the vessels’ passive collapse, which reduces CBF, CPP, and increases oxygen extraction from hemoglobin, but for CBF below 20 mL/100 g/min the brain becomes ischemic. Likewise, secondary injuries can also initiate a “vicious cycle” (a term widely described in the literature in this field [12,66,89,90]), where increased intracranial volume raises intracranial hypertension, which in turn leads to arterial hypertension as a compensatory response to maintain CPP (cushing response) [91]. However, under autoregulatory failure conditions, the latter response increases CBF, which exacerbates secondary brain injury (Figure 11) [91].

##### Summary of the Evidence on NIRS-derived Autoregulation in TBI

The literature search returned nine scientific publications that assessed NIRS derived autoregulation in TBI patients. Table 2 summarizes the relevant data extracted from each document. The papers were sorted chronologically by publication year, as is presented in the first column of the table along with the author. Moreover, sample size (*n*) is reported with the proportion of male and female (m:f) included in the studies. Column three of Table 2 includes the NIRS devices used, the manufacturer, and some technical specifications in brackets. Furthermore, NIRS outputs and control measurements are shown in columns four and five. Only in one case the comparison was between TBI patients and healthy control volunteers. Finally, the main conclusion of each paper is shown in the last column of the table.

##### Sample Size and Patient Demographics

**Figure 12:**
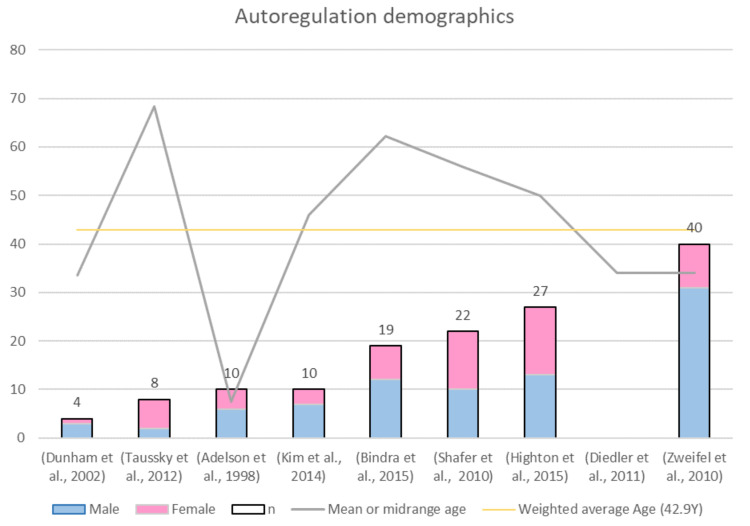
Demographics of the evidence on NIRS-derived autoregulation in TBI.

This review identified nine documents spanning between 1998 and 2015 where NIRS was used for autoregulation monitoring in TBI patients. Figure 12 shows that only 25% of the studies had a sample size larger than 27 patients, which is a limitation in the results of almost all these publications. As aforementioned, men being at more than double the risk of TBI than women [2], which agrees with the weighted average of the sex ratios (m/f = 1.9). Finally, the mean or midrange age is presented in Figure 12 with a grey colour line. One outlier in the age dataset can be noticed in the figure, and this is because this study by Adelson et al. assessed TBI pediatric patients [86]. Therefore, age median is 46 years, and its interquartile range is between 34 and 56 years. Interestingly, all studies included mostly middle-aged people, who are at risk of suffering severe TBI but can also be rapidly stabilized in intensive care.

##### Distribution of NIRS Techniques

**Figure 13:**
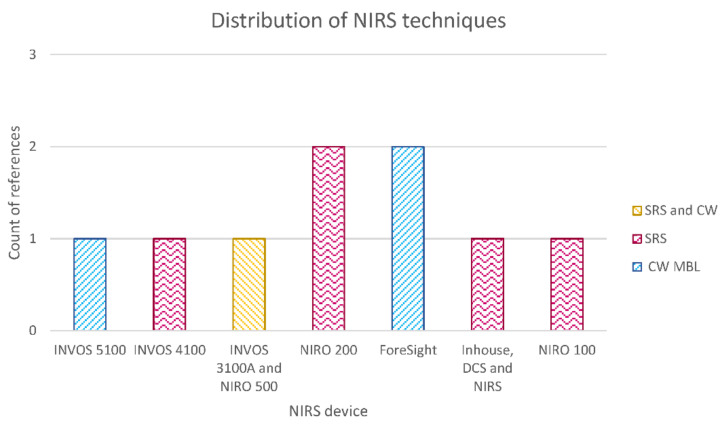
Distribution of NIRS techniques used for autoregulation monitoring in TBI patients.

Figure 13 shows the distribution of NIRS techniques used for autoregulation monitoring in TBI patients. Similarly, with the studies on oxygenation, the two most used techniques for autoregulation assessment were continuous wave (CW) NIRS and spatially resolved spectroscopy (SRS). Only three papers reported the source–detector (S-D) distance, having values between 2.5 and 4.5 cm in reflectance mode [96,97,98]. The following three brands were repetitively used in cerebral autoregulation papers: NIRO monitor from Hamamatsu Photonics, INVOS system from Medtronic, and ForeSight Oximeter from Casmed. However, the device model changed between authors. An inhouse hybrid system was used in one of the included publications. Kim et al. presented a custom-built device containing both diffuse correlation spectroscopy (DCS) and NIRS. DCS is a novel optical technique for probing continuous changes in regional microvascular blood flow, while NIRS was used for oxygenation parameters’ changes. Together, both techniques were used to detect differences in cerebral hemodynamic responses of brain-injured patients to posture change [97].

##### Appropriate Clinical Comparable Parameter

**Figure 14:**
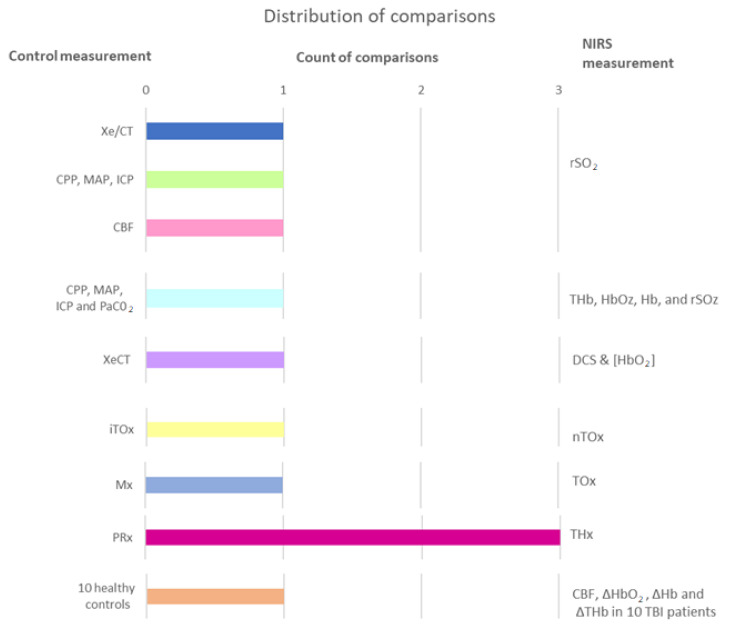
Comparisons found in the evidence on NIRS-derived autoregulation in TBI.

This section presents the different comparisons found in the literature to assess the capability of NIRS in cerebral autoregulation monitoring. Comparisons are divided into three types: (1) When NIRS-derived values are associated with physiological measurements, (2) when the clinical comparable parameter is an index, and (3) when TBI patients are compared to healthy controls. The latest relates specifically to Kim’s paper, where two cohorts were exposed to postural changes in order to identify whether a noninvasive in-house system is able to detect CBF and hemoglobin changes (orange bar of Figure 14) [97].

In the first type are the comparisons made between NIRS variables, such as rSO_2_ or the concentration of different forms of hemoglobin, and physiological parameters that are measured invasively or through tomographic images. As it was explained in section “Physiology of Autoregulation in TBI”, the main variables involved in cerebral autoregulation are CBF, CPP, MAP PaCO_2_, and ICP. In consequence, it is not surprising that some authors aim to associate NIRS outcomes with these comparators [86,92,94,96].

In the second type, the comparisons are made between invasive and noninvasive indexes. The most frequent observation is between pressure reactivity index (PRx) and its noninvasive analogous total hemoglobin reactivity index (THx), as it is shown by the fuchsia column in Figure 14 [93,98,100]. The pressure reactivity index is defined as the moving correlation between MAP and ICP. When cerebrovascular reactivity is impaired, intracranial volume (ICV) and ICP increase and decrease passively with MAP. Thus, a negative value for PRx, when ICP is inversely correlated with MAP, indicates normal reactivity, and a positive value indicates impaired autoregulation [93,98]. Likewise, THx is calculated as the moving correlation between the total hemoglobin index ($\mathrm{THI}=\left[{\mathrm{HbO}}_{2}\right]+\left[\mathrm{HHb}\right]$) and arterial blood pressure (ABP) [100].

Other comparisons between indexes were presented by Highton et al., who compared the mean velocity index (Mx) with the tissue oxygen reactivity index (TOx). Interestingly, both indexes are obtained using noninvasive techniques. For instance, Mx is derived from transcranial Doppler-measured flow velocity and ABP, while TOx is the correlation between NIRS-derived rSO_2_ and ABP. However, arterial blood pressure was measured invasively using a 20-G radial cannula and a transducer placed at the level of the tragus [98]. Unlike Highton’s study, Brinda et al. compared tissue oxygen reactivity indexes calculated as the moving correlation coefficient between invasive ABP and rSO2 (iTOx), and the moving correlation coefficient between noninvasive ABP and rSO2 (nTOx). The latter used a Finometer photoplethysmograph (Finometer pro, Finapres Medical Systems, Netherlands) for noninvasive arterial blood pressure monitoring [99].

## 4. Discussion

This review provides readers with a comprehensive summary of the technological advancements in the field of NIRS for TBI monitoring. This analysis not only considered applications for TBI, but also investigated the use on NIRS in different patients’ demographics, and described the most-used techniques and testing clinical comparable parameters.

**Figure 15:**
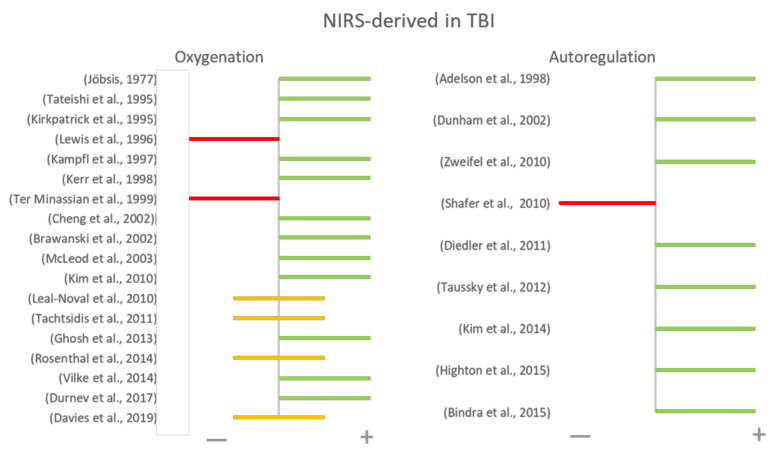
Main conclusions of NIRS-derived monitoring in TBI. Each author concluded with a positive (green) or negative (red) statements. In a few cases, authors reported positive results with some limitations (yellow).

Figure 15 summarizes the final conclusions from all reviewed papers on the suitability of NIRS in monitoring oxygenation or autoregulation in TBI patients. Positive results are defined as results where the comparison between NIRS and gold standard techniques yielded high correlations or agreements, and negative results where such correlations were not high. Figure 15 provides a general representation of the results presented by each author, despite the heterogeneity in their hypothesis, methods, and outcomes. Additionally, the chart depicts the risk of publication bias, where papers with positive results are more likely to be published by indexed journals than those which rejected their hypothesis.

Regarding the application of NIRS in oxygenation monitoring, patients’ samples were homogeneous amongst the evidence. However, there is still a lot of variation in NIRS techniques and the reference measurements, which decreased the overall comparability between the results. Technical parameters such as the algorithm used within NIRS, wavelengths, and source–detector(s) separation differ between the commercially available devices, making it difficult to draw robust comparisons between them. All the papers which assessed paired oxygenation measurements reported positive results, which can be considered as a significant change on NIRS signals before and after an intervention (change on MAP, hyperoxia, hypercapnia, etc.). The interventions aim to disrupt normal cerebral hemodynamics, so significant changes on NIRS outcomes strengthens the belief that these are derived primarily from cerebral tissues and are highly correlated with brain hemodynamic changes [21,68,73,74,77]. For example, papers related to oxCCO monitoring reported that changes in concentration during normobaric hyperoxia represent an actual increase in mitochondrial aerobic metabolism [73,74]. On the other hand, in those cases where NIRS-derived oxygenation parameters were compared with variables that are not directly related to oxygenation, such as ICP, CBF or mortality, the authors also reported positive findings [65,76,97]. For instance, rSO_2_ values in intracranial hypertensive patients were significantly lower than in nonhypertensive patients [65]. Likewise, DCS cerebral blood flow measurements had a moderate correlation with rSO_2_ [97]. Finally, rsO2 was more accurate in discriminating and predicting hospital mortality than the traditional clinical parameters [76].

Conversely, studies that compared NIRS measurements with invasive oxygenation monitoring techniques, such as SjO_2_ and PbtO_2_, had heterogeneous results. Hemoglobin concentration measured by NIRS was correlated with SjO_2_ [62,63]. It was found that NIRS detected visible changes in the chromophores levels when desaturation events occurred, even with twice the sensitivity of SjO2, which might be explained by the fact that SjO2 sensitivity is affected by the venous blood that is drained from the sinuses to the jugular veins [63]. Nonetheless, the agreement between regional tissue oxygen saturation and SjO_2_ was significant in only a few papers [64,66,67,70,75]. Similarly, rSO_2_ and PbtO_2_ contain similar information from a mathematical point of view, but NIRS outcomes have not demonstrated enough reproducibility in its ability to predict changes in PbtO_2_ in order to replace this invasive measurement [69,72,78]. The technological heterogeneity between the papers due to the use of CW-MBL or SRS may explain the contradictory nature of the data.

On the other hand, papers that used NIRS in autoregulation monitoring reported generally positive conclusions despite the various methodological and technological limitations of NIRS. For instance, some papers where NIRS-derived values were compared with physiological measurements found significant correlations. Dunham et al. reported an association between rSO_2_ levels above 75% and CPP ≥ 70 mmHg, while Taussky et al. found a correlation between pairs of values of rSO_2_ and CBF [92,96]. However, Shafer et al. reported no significant correlation between the above relationship and mentioned that an expansion of the study to a greater number of patients may uncover such a relationship [94]. It is interesting that both authors Taussky et al. (*n* = 8) and Shafer et al. (*n* 22) compared rSO_2_ measurements with CT-derived CBF and had such conflicting results. However, these conflicting results might be due to methodological heterogeneities, including differences in the NIRS devices used [94,96]. The last type 1 comparison (when NIRS-derived values are associated with physiological measurements) showed that during hyperventilation, ICP, CPP, and PaCO_2_ correlated with total hemoglobin concentration measured by NIRS. As it is known, during hyperventilation, decreased PaCO_2_ results in cerebral oxygen desaturation, regardless of lCP levels. Then, NIRS positively predicted cerebral oxygen desaturation and indicated increased total hemoglobin (relative CBV) [86]. These results are encouraging, suggesting that NIRS can be used as a noninvasive monitoring method for the earlier recognition and subsequent treatment of TBI secondary insults.

Likewise, the agreement between indexes was assessed successfully by several authors [93,98,99,100]. Most of these papers used the NIRO systems from Hamamatsu Photonics and reported significant a correlation between THx and PRx [93,98]. Diedler et al. suggested that this agreement is a function of the power of slow oscillations in the input signals [100]. Moreover, Highton also found significant agreement between TOx-NIRS index and Mx-TCD index [98]. However, THx and TOx were not completely noninvasive indexes, as they depended on the continuous invasive monitoring of MAP [93,98]. In order to respond to this limitation, Bindra et al. calculated MAP using a Finometer photoplethysmography system and found similarities between partially invasive TOx and completely noninvasive TOx [99]. Likewise, Kim et al. tested their hybrid DCS/NIRS instrumentation in TBI patients and healthy controls [98]. The aforementioned paper suggested that future studies in cerebral autoregulation should use multimodal monitoring combining NIRS analysis of indexes, such as THx and TOx.

There is still a need for more research in NIRS-derived monitoring in TBI patients. Current results are limited due to the small number of subjects evaluated. Studies with representative sample size are needed in order to have inferable results in the TBI population. Despite the positive results that NIRS showed in these research studies, there is still more work to be done in comprehensively evaluating NIRS in order to be established as a reliable and routine monitoring technique in TBI. Considering that brain oxygenation and autoregulation are often measured in severe TBI patients, it is not surprising that the studies included in this review investigated populations of severe TBI patients only. However, most traumatic brain injuries are categorized as mild to moderate, and future research on NIRS application would greatly benefit these patients who often do not receive brain oxygenation or autoregulation monitoring [101].

Moreover, high metrological heterogeneity decreased the possibility of an overall conclusion, even between studies that utilized the same NIRS technique. Also, it is important to define a valid clinical comparable parameter which reflects tissue brain oxygenation and autoregulation, as the current invasive references utilize distinct physical principles and measure distinct physiological parameters. For instance, SjO_2_ and PbtO_2_ techniques provide complementary information reflecting different aspects of the cerebral oxygenation cascade, but do not represent the regional tissue oxygen saturation. Likewise, a standardized clinical comparable parameter for autoregulation monitoring in neuro-critical care centers is still missing, which may be due to the lack of dedicated autoregulation monitors [81].

Additionally, the global outcome measures were not uniform across all studies, therefore, for future purposes of quantitative synthesis of the evidence, it is recommended that the authors report Bland–Altman analysis. It is a simple and accurate way to assess the agreement between two clinical variables and may help clinicians to compare a new measurement method against a standard reference [102]. Table 3 summarizes some strengths and limitations of this technology as reported in the papers included in this review. The limitations presented below are highly related to the lack of standardization of NIRS devices and some to patient-related factors.

The main strength of this review is that it presents a synthesis of NIRS applications specifically for TBI patient monitoring. Additional challenges are related to measuring light tissue interaction changes after TBI, due to alterations in the pathophysiological process and damage in post-traumatic tissue. The latter makes it difficult to extrapolate NIRS results in normal tissue to TBI patients with the possible presence of extracranial blood, subdural air, hemorrhages, cerebral edema, and some other clinical scenarios. However, TBI as an inclusion criterion restricted the number of studies included in this review. Likewise, no attempts were made to identify or translate non-English language publications, and this may have limited the inclusion of some relevant studies in this review. Also, publication bias may have occurred, because only peer-reviewed literature was included, and public health reports on NIRS monitoring in TBI may be available in the gray literature. However, these criteria provided the added ability to focus on the results in the literature in line with NIRS technological advancements in TBI monitoring.

## 5. Conclusions

This review is a synthesis of the characteristics and advantages of NIRS use in TBI and the available evidence of the association between continuous NIRS-based measures and commonly monitored neurophysiological parameters, such as oxygenation and cerebral autoregulation. So far, evidence is primarily focused on monitoring adults with severe TBI, where CW and SRS are the most-used NIRS technologies. However, the methodological approaches between studies remain heterogenous. The above might be due to the lack of “dedicated autoregulation monitors”; likewise, the comparison of oxygenation gold standards with NIRS-derived paraments is problematic, as each monitor utilizes a distinct physical principle and measures a distinct physiological parameter. As oxygenation and autoregulation seem to play a crucial role in patient treatment and outcome, the potential use of NIRS in combination with multimodal monitoring is in the best interest of both TBI patients and clinicians.

## Figures and Tables

**Table 1:** Summary of the evidence on NIRS-derived oxygenation in TBI.

Author, Year	N (m:f)	Device (Type)	Output	Control	Conclusion
(Jöbsis, 1977) [21]	1 (1:0)	Inhouse NIRS, non commercial (CW; S-D 13.3 cm)	Photon count	Paired measure: hyperventilation	Oxygen sufficiency can be monitored noninvasively.
(Tateishi et al., 1995) [62]	9 (9:0)	NIRO-500, Hamamatsu Photonics, Hamamatsu, Japan. (CW; S-D 4 cm)	HbO_2_	SjO_2_	Cerebral HbO_2_ magnitude and direction, measured by NIRS, were similar to changes in invasive measurements of SjvO_2_.
(Kirkpatrick et al., 1995) [63]	14 (12:2)	NIRO 1000, Hamamatsu Photonics U.K. Ltd., Enfield, UK. (CW; S-D 6 cm)	HbO_2_	CPP, ICP and CBFSjO_2_	A close correlation between NIRS signals and intracranial parameters strengthens the belief that the observed chromophore concentration changes are derived primarily from cerebral tissues.
(Lewis et al., 1996) [64]	10 (6:4)	INVOS 3100, Medtronic, Minneapolis, MN, USA. (SRS; S-D 3&4 cm)	rSO_2_	SjO_2_	Tissue oxygen saturation determined by near-infrared spectroscopy does not reflect significant changes in cerebral oxygenation detected by the global measurement of jugular venous bulb oximetry.
(Kampfl et al., 1997) [65]	8 (5:3)	INVOS 3100A, Medtronic, Minneapolis, MN, USA. (SRS; S-D 3&4 cm)	rSO_2_	ICP	rSO_2_ values in patients with an ICP > 25 mmHg were significantly lower than in patients with an ICP < 25 mmHg after the hyperoxygenation period.
(Kerr et al., 1998) [66]	28 (25:3)	INVOS 3100A, Medtronic, Minneapolis, MN, USA. (SRS; S-D 3&4 cm)	rSO_2_	SaO_2_, SjvO_2_, extracranial SO_2_	rSO_2_ index represented a summation of differential weighting of SaO_2_ (20%), SjvO_2_ (75%), and extracranial O_2_ saturation (5%).
(Ter Minassian et al., 1999) [67]	9 (NR)	INVOS 3100, Medtronic, MN, USA. (SRS; S-D 3&4 cm)	rSO_2_	SjO_2_	rSO_2_ assessed by NIRS does not adequately reflect changes in SjvO_2_ in patients with a severe head injury.
(Cheng et al., 2002) [68]	9 (7:2)	CCD detector system, noncommercial John Wright, UK. (CW; S-D 3.5 cm)	HbO_2_ oscillations	NR	The presence of oscillations at 0.013–0.033, 0.11, and 0.19–0.28 Hz are compatible with B-waves, vasomotion, and respiratory cycles, respectively.
(Brawanski et al., 2002) [69]	12 (11:1)	INVOS 3100, Medtronic, Minneapolis, MN, USA. (SRS; S-D 3&4 cm)	rSO_2_	PbtO_2_	rSO_2_ and PbtO_2_ contain similar information from a mathematical point of view.
(McLeod et al., 2003) [70]	8 (8:0)	NIRO 300, Hamamatsu Photonics, Hamamatsu City, Japan. (SRS; S-D 4 cm)	TOI	SjO_2_ and PbtO_2_	Altering the fraction of inspired oxygen changes significantly each variable measured of cerebral oxygenation. Each variable represents a different physiologic process.
(Kim et al., 2010) [71]	8 (5:3)	Inhouse, noncommercial (CW; S-D 2.5 cm)	DCS and HbO_2_	XeCT	Significant moderate correlations between DCS measurements of relative CBF and NIRS measurements of delta HbO_2_ were demonstrated.
(Leal-Noval et al., 2010) [72]	22 (NR)	INVOS 5100, Medtronics Inc., MI, USA. (CW; NR)	rSO_2_	PbtO_2_	PbrO_2_ and rSO_2_ were directly and significantly related. However, the diagnostic accuracy of rSO_2_ was limited, therefore, that measurement by NIRS should not be considered to be an acceptable substitute for PbrO_2_.
(Tachtsidis et al., 2011) [73]	6 (5:1)	Broadband NIRS, noncommercial (CW; S-D 3.5 cm)	oxCCO	Paired measure: hypercapnia	Despite the increase in total HbO_2_ in all patients, only four of the six patients showed an increase in the oxidation states.
(Ghosh et al., 2013) [74]	10 (3:7)	Inhouse, noncommercial (SRS; S-D 3.5 cm)	oxCCO	Paired measure: normobaric hyperoxia	Optical measurement of chromophore concentration in the injured brain is not confounded by changes in optical scattering or pathlength.
(Rosenthal et al., 2014) [75]	18 (13:5)	CerOx 3110, Ornim Medical Ltd. Dedham, MA, USA. (UT-NIRS; NR)	rSO_2_	SjO_2_ PbtO_2_	The correlation between UT-NIRS measurements and SjvO_2_ indicate that the CerOx may be able to provide a noninvasive estimation of cerebral oxygenation status in brain-injured patients. However, rSO_2_ was not correlated with PbtO2.
(Vilke et al., 2014) [76]	61 (42:19)	INVOS, Medtronics Inc., MI, USA. (SRS; NR)	rSO_2_	Mortality	rSO_2_ values were determined as a strong discriminator and predictor of hospital mortality. When rSO_2_ < 68.0% in the left hemisphere HR = 17.7.
(Durnev et al., 2017) [77]	15 (10:5)	INVOS 5100, Medtronics Inc., MI, USA. (CW; NR)	rSO_2_	Paired measure: changes on MAP	NIRS signals of cerebral hypoxygenation reacted first to MAP changes.
(Davies et al., 2019) [78]	16 (9:3)	Oxiplex TS, ISS, Il, USA. (PMS; NR)	rSO_2_	PbtO_2_	A clear predictive relationship between NIRS and invasively measured PbtO_2_ has been established. However, FD enhances NIRS device tested did not demonstrate sufficient reproducibility in its ability to predict changes in PbtO2 to replace the current invasive gold standard.

Abbreviations: regional tissue oxygen saturation (rSO2), hemoglobin concentration (HbO_2_), jugular bulb venous saturation (SjO_2_), cerebral perfusion pressure (CPP), intracranial pressure (ICP), cerebral blood flow (CBF), arterial oxygenation (SaO_2_), brain tissue oxygen tension (PbtO_2_), xenon-enhanced computed tomography (XeCT), mean arterial pressure (MAP), hazard ratio (HR), frequency domain (FD), near-infrared spectroscopy (NIRS), cytochrome-c-oxidase concentration (oxCCO), continuous wave modified Beer–Lamber law (CW), spatially resolved spectroscopy (SRS), phase modulated spectroscopy (PMS), ultrasound-tagged near-infrared spectroscopy (UT-NIRS), source detector distance (S-D), not reported (NR).

**Table 2:** Summary of the evidence on NIRS-derived autoregulation in TBI.

Author, Year	N (m:f)	Device (Type)	Output	Control	Conclusion
(Adelson et al., 1998) [86]	10 (6:4)	INV03100A, Medtronic, MN, USA and NIRO500, Hamamatsu Photonics, Hamamatsu, Japan. (SRS and CW respectively; S-D NR)	THb, HbO_2_, Hb, and rSO_2_	CPP, MAP, ICP and PaCO_2_	High ICP and decreased CPP correlated with increased THb and HbO_2_ indicating raised CBV and hyperemia. MAP was not associative. NIRS positively predicted cerebral oxygen desaturations with hyperventilation.
(Dunham et al., 2002) [92]	4 (3:1)	INVOS 4100, Medtronic, MN, USA. (SRS; S-D NR)	rSO_2_	CPP, MAP, ICP	Cerebral oximetry correlated significantly with CPP. As such, it could be an adjunct to CPP management.
(Zweifel et al., 2010) [93]	40 (31:9)	NIRO 200, Hamamatsu Photonics U.K. Ltd., Hertfordshire, UK. (SRS; S-D NR)	THx	PRx	THx showed a significant correlation with the validated volume reactivity index PRx.
(Shafer et al., 2010) [94]	22 (10:12)	INVOS 5100, Medtronic, MN, USA. (CW; S-D NR)	rSO_2_	XeCT	The relationship between either the left or right NIRS values and Xe/CT scan was not significant.
(Diedler et al., 2011) [95]	37 (NR)	NIRO 200, Hamamatsu Photonics U.K. Ltd., Hertfordshire, UK. (SRS; S-D NR)	THx	PRx	The agreement between PRx and THx is a function of the power of slow oscillations in the input signals.
(Taussky et al., 2012) [96]	8 (2:6)	Bifrontal NIRS optodes, Casmed, Branford, CT, USA. (CW; S-D 4.5 cm)	rSO_2_	CBF	CT perfusion CBF has a significant linear correlation with NIRS derived rSO_2_.
(Kim et al., 2014) [97]	10 (7:3)	Inhouse, DCS and NIRS system, Noncommercial. (SRS; S-D 2.5 cm)	CBF, ΔHbO_2_, ΔHb and ΔTHb in 10 TBI patients	CBF, ΔHbO_2_, ΔHb and THb in 10 healthy controls	HbO2, Hb, and THb concentration increased significantly in the brain-injured cohort with head-of-bed lowering. Accordingly, DCS/NIRS hybrid device is well-suited to provide non- invasive, continuous hemodynamic monitoring.
(Highton et al., 2015) [98]	27 (13:14)	NIRO 100, Hamamatsu Photonics U.K. Ltd., Hertfordshire, UK. (SRS; S-D 4 cm)	THx, TOx	PRx, Mx	Significant agreement among PRx and THx, and between Mx and TOx. However, the strength of the interrelationship between ICP or TCD and NIRS signals, THI or rSo2, limits the degree of agreement between these reactivity indices.
(Bindra et al., 2015) [99]	19 (12:7)	ForeSight, Casmed, Connecticut, USA. (CW; S-D NR)	nTOx	iTOx	nTOx from Finometer photoplethysmography and NIRS gives a similar measurement of cerebrovascular autoregulation to iTOx.

Abbreviations: regional tissue oxygen saturation (rSO_2_), oxyhemoglobin concentration (HbO_2_), total hemoglobin concentration (THb), deoxyhemoglobin concentration (Hb), concentration change (Δ), cerebral perfusion pressure (CPP), intracranial pressure (ICP), cerebral blood flow (CBF), partial pressures of arterial carbon dioxide (PaCO_2_), xenon-enhanced computed tomography (XeCT), mean arterial pressure (MAP), pressure reactivity index (PRx), total hemoglobin reactivity index (THx), tissue oxygen reactivity index (TOx), mean velocity index (Mx), noninvasive tissue oxygen reactivity index (nTOx), invasive tissue oxygen reactivity index (iTOx), near-infrared spectroscopy (NIRS), continuous wave modified Beer–Lambert law (CW), spatially resolved spectroscopy (SRS), source detector distance (S-D), not reported (NR).

**Table 3:** Strengths and limitations of NIRS-derived oxygenation in TBI.

**STRENGTHS OF NIRS** Effective transmission of NIR light in biological tissue allows non-invasive monitoring of cerebral HbO_2_ and HHb, blood volume, the redox state of cytochrome, and thereby cerebral oxygen sufficiency [21]. Near-infrared spectroscopy findings demonstrated visible changes in HbO_2_ and Hb levels in approximately twice as many desaturation events as those registered with SjO_2_ monitoring [63]. The close correlation between NIRS signals and those derived from known intracranial parameters strength-ens the belief that the observed chromophore concentration changes are derived primarily from cerebral tissues [63]. NIRS may be a valuable tool in the detection of impaired microcirculation and/or local brain tissue oxygena-tion in patients with increased intracerebral pressure, which may not be detectable employing monitoring of CPP, blood gas analysis, and TCD velocities [65]. The rS0_2_ index provides an easy, noninvasive method to measure decrements in oxygen delivery or utilization of O_2_ within the brain [66]. NIRS is able to provide a clinically accessible, continuous, and noninvasive measure of cerebral hemodynamic and therefore has considerable potential as a noninvasive monitor of cerebral autoregulation [98].
**LIMITATIONS OF NIRS** Penetration of light is limited to several centimetres in-depth, and the precise sampling volume and site of measurement are not fully clear [62]. Hemoglobin in vessels of superficial structures may add extracerebral signals, and hence adding interference to CW-NIRS measurements [62]. Bulky, sensitive to variations in room temperature, and exquisitely sensitive to outside light [63]. The signals also display drift and are sensitive to movement artifact [63]. Alterations in cerebral blood flow and metabolism following severe head injury are heterogeneous, and re-gional differences measured by NIRS may not be reflected by a global measurement such as jugular venous bulb oximetry [64]. The rS0_2_ index reflects a regional measure, while the jugular venous gas analysis is global [66]. The pathlength factor and the depth concerning head swelling following trauma make the site of measure-ment and the volume sample ambiguous [66]. The cerebral signal could be contaminated by a reflected signal from extracerebral structures (e.g., bone, mus-cle) with unpredictable partition and O_2_ saturation characteristics [67]. The clinical use of NIRS remains limited by potential sources of error that include contamination of the signal by the extracerebral circulation (principally the scalp), extraneous light, and the presence of extravascular blood [70]. Hematomas may prevent sufficient photon transmission through the cortex due to excessive absorption by the concentrated blood [71]. Edema directly beneath the optical probes may also prevent signal detection when light absorption is high [71]. In NIRS studies, many severe TBI patients are excluded from data analysis because of intra- and extracranial problems [72]. Other patient-related factors affecting the NIRS-derived values include patient agitation, skin conditions (burn, infection, or scar), brain malformation, polycythemia, and subcutaneous fat [54].
